# Molecular basis of the different effects of procainamide and N-acetylprocainamide on the maximum upstroke velocity and half-decay time of the cardiac action potential in guinea pig papillary muscle

**DOI:** 10.1590/1414-431X2023e12073

**Published:** 2023-01-27

**Authors:** W. Sigler, A.C. Oliveira

**Affiliations:** 1 Departamento de Farmacologia, Instituto de Ciências Biomédicas, Universidade de São Paulo, São Paulo, SP, Brasil; 2 Faculdade de Ciências Farmacêuticas e Bioquímicas, Faculdades Oswaldo Cruz, São Paulo, SP, Brasil

**Keywords:** Procainamide, N-acetylprocainamide, Cardiac action potential, Maximum upstroke velocity, Half-decay time

## Abstract

Procainamide (PA) and its *in vivo* metabolite, N-acetylprocainamide (NAPA), display some pharmacological differences. Although it is agreed that PA is a class IA antiarrhythmic, it has been reported that NAPA is a pure class III antiarrhythmic that affects only the repolarizing phase of the cardiac action potential. This last concept, observed exclusively in dogs, gained wide acceptance, appearing in classic pharmacology textbooks. However, evidence in species such as mice and rats indicates that NAPA can affect cardiac Na^+^ channels, which is unexpected for a pure class III antiarrhythmic drug. To further clarify this issue, the effects of PA (used as a reference drug) and NAPA on the maximum upstroke velocity (Vmax) and half-decay time (HDT) of the cardiac action potential were examined in the isolated right *papillaris magnus* of the guinea pig heart. Both PA and NAPA affected Vmax at lower concentrations than required to affect HDT, and NAPA had weaker effects on both variables. Thus, NAPA displayed typical class IA antiarrhythmic behavior. Therefore, the concept that NAPA is a pure class III antiarrhythmic drug is more species-dependent than previously envisioned. In addition, we demonstrated that the differential pharmacology of PA and NAPA is explainable, in molecular terms, by steric hindrance of the effects of NAPA and the greater number of potent aromatic-aromatic and cation π interactions with Na^+^ or K^+^ cardiac channels for PA.

## Introduction

Procainamide (PA) is classified as a class IA antiarrhythmic drug because it decreases the maximum upstroke velocity (Vmax) of the cardiac action potential at lower concentrations, and increases the half-decay time (HDT) of the cardiac action potential at higher concentrations (1,2).

*In vivo*, PA is metabolized to the active metabolite N-acetylprocainamide (NAPA) (3), which is generally considered a pure class III antiarrhythmic drug in pharmacology textbooks (1,2). This concept has arisen because previous electrophysiological studies demonstrated that NAPA increases the HDT of the cardiac action potential without affecting Vmax (4-7). However, only a single animal species, namely dogs, was used in all of these studies (4-7), and it is well known that the pharmacology of cardiac antiarrhythmic drugs is highly species-dependent (8). Indeed, some data regarding NAPA generated in other species do not agree with the concept that NAPA is a pure class III antiarrhythmic drug. Accordingly, we obtained direct evidence that NAPA blocks Na^+^ channels in isolated mouse cardiac myocytes (9). It should be noted that Na^+^ channel blockade is not expected for a pure class III antiarrhythmic drug which, by definition, should only block cardiac potassium channels and consequently should only affect HDT (1,2).

**Figure 1:**
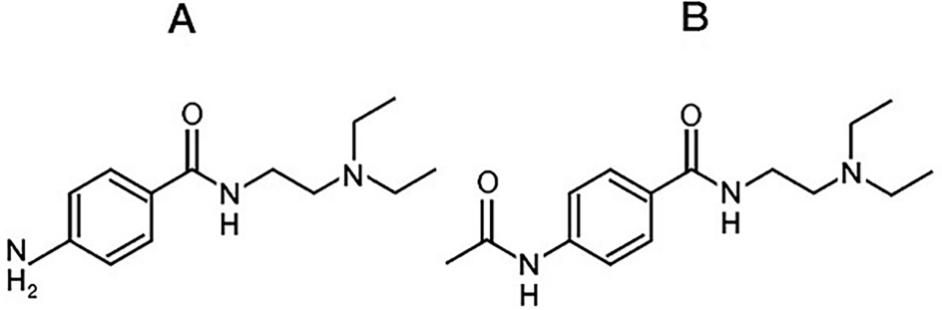
Chemical structures of procainamide (**A**) and N-acetylprocainamide (**B**).

Furthermore, in our previous study (9) we compared NAPA with PA (used as a reference drug) and proved that the former was less potent. This observation was unusual because NAPA is more lipophilic than PA (4), and it is thus expected to be more potent than PA. This is because both PA and NAPA have the chemical structure of local anesthetics, namely an aromatic moiety at one end of the molecule connected by an alkyl chain to an ionizable amino group at the opposite end of the molecule (Figure 1A and B). Generally, the potency of local anesthetics varies positively with lipophilicity (10-12).

To the best of our knowledge no study provided an explanation on a molecular basis regarding the ratio of potencies favoring PA over NAPA, despite the higher lipophilicity of the latter. Thus, there is clearly a need to comprehensively reevaluate the cardiac pharmacology of NAPA by encompassing two successive and complementary steps.

First, an electrophysiological/pharmacological study of the recorded intracellular action potential should be performed using a larger spectrum of NAPA concentrations than previously reported in the literature (4-7), and NAPA should be compared with a reference drug (PA in our case). The use of a reference drug proved to be an important item in the design of comparative studies involving antiarrhythmic drugs (13). The cardiac action potential should be scrutinized with respect to Vmax and HDT, because Vmax reflects the status of cardiac Na^+^ channels and HDT reflects the status of cardiac K^+^ channels. This strategy should permit the comprehensive evaluation of the effects of either PA or NAPA on cardiac Na^+^ and K^+^ channels.

The second step is collating the obtained comparative electrophysiological/pharmacological effects of PA and NAPA on the action potential to the known molecular mechanisms of Na^+^ or K^+^ channel blockade described in the literature. These mechanisms involve different types of interaction, i.e., hydrophobic, aromatic-aromatic, and cation-π interactions, for both Na^+^ (14-19) and K^+^ (20-24) channel blockers. Because these interactions are modulated by diverse physicochemical parameters that encode lipophilicity and electronic and steric properties of molecules, such parameters should therefore be comparatively studied for PA and NAPA. Because no study has examined these variables, we have done so in the present work.

## Material and Methods

### Preparation

Dunkin-Harley guinea pigs of both sexes (weight 300-500 g) were used in the experiments. The animals were initially subjected to general anesthesia using a mixture of ketamine (200 mg/kg) and xylazine (60 mg/kg) administered intraperitoneally (25). With the animals under general anesthesia, thoracotomy was performed. These procedures were performed in accordance with the AVMA guidelines for the euthanasia of animals (26) and endorsed by the Committee on Ethics in Animal Experimentation of the Institute of Biomedical Sciences, University of São Paulo (Brazil). Upon thoracotomy, the heart was quickly removed. The *papillaris magnus* muscle was excised from the hearts and pinned to a layer of transparent Sylgard^®^ resin covering the bottom of a 20 mL chamber. Warm water circulating through a jacket surrounding the chamber kept the bathing solution at a constant temperature of 37.0±0.5°C. The preparation was bathed in Tyrode's solution with the following composition: NaCl: 135 mM; KCl: 5 mM; MgCl_2_: 1 mM; CaCl_2_: 2 mM; NaHCO_3_: 15 mM; Na_2_HPO_4_: 1 mM; glucose: 11 mM. The solution was continuously gassed with a mixture of O_2_ (95%) and CO_2_ (5%). The pH of Tyrode's solution ranged from 7.3-7.4.

### Drugs

PA was obtained from Sigma-Aldrich (USA). NAPA was obtained from Sigma-Aldrich or Dr. Maria Auxiliadôra Fontes Prado, from the Federal University of Minas Gerais, Brazil.

### Electrophysiology

A detailed description of the equipment and procedures used in this laboratory in the electrophysiological approach was previously reported (27). Briefly, action potentials were evoked by stimulating the preparation using a small bipolar platinum electrode connected to an electronic stimulator via a stimulus isolation unit. Membrane resting and action potentials were recorded using conventional intracellular microelectrode techniques. Resting membrane potentials were recorded in the DC mode and action potentials were recorded in the AC mode. The recorded AC data were amplified 20 times, low pass filtered (10 kHz) and stored in digital form (50 kHz sampling rate) using an analog-to-digital converter (Lynx, Brazil) coupled to a microcomputer. The analog-to-digital converter was commanded by a software (AqDados 7.5, Lynx, Brazil) that propitiated the sampling and filing of the action potentials, on line, and retrieved the stored waveforms, off line. The software also permitted the manipulation, using cursors, of the retrieved digitized action potentials to measure electrophysiological variables of interest, which were: Vmax, (maximum upstroke velocity of the action potential) and HDT (the time taken for the action potential to repolarize to 50% of its peak value).

Vmax was obtained as follows. The stored digitized action potential recording was retrieved, and its rising phase was scrutinized, using cursors, to determine the steepest slope within any two vicinal points. In this region, the change in voltage between these two points was maximal throughout the whole rising phase of the action potential. Vmax was calculated as the quotient between this maximal value and the corresponding time required by the waveform to travel between the two points.

HDT of the action potential was also obtained from the stored digitized recording using cursors. It was defined as the time for the waveform to fall to 50% of the maximal amplitude value.

To perform the electrophysiological experiments, control recordings were initially obtained in cells bathed in a drug-free physiological solution. Next, the same muscle was bathed (at least for 30 min) with a physiological solution containing either PA or NAPA. Action potentials were generated following a resting time of 1-3 min, during which the muscle was permitted to rest, i.e., it was not stimulated. In view of this protocol, the “resting” conformations of both the Na^+^ and K^+^ cardiac channels constituted the targets for the drugs.

### Physicochemical parameters of PA and NAPA

Lipophilicity and electronic and steric physicochemical parameters (28,29) were used to disclose the molecular basis modulating the effects of PA and NAPA.

The lipophilicity parameters were as follows: log P (the logarithm of the octanol/water partition coefficient); log D (the logarithm of the lipid distribution coefficient: log D = log P- (1+ 10^(pKa-pH)^), and π (descriptor of the lipophilicity of the substituent in the benzene ring). Log P and log D were calculated using a software (30), whereas π was obtained from the literature (28,29).

The electronic parameters were pKa and Hammett's constant of aromatic p-substituents (σ_p_), both obtained from the literature (28,31,32). The steric parameters quantified the size of the substituents. To this end, the following steric parameters calculated using the STERIMOL program (28,29,33) were used: a) the L parameter, which is a measure (Å) of the length of the substituent; and b) B_1,_ B_2,_ B_3_, and B_4_, which are measures (Å) of the width of the substituents, in order from the smallest value (B_1_) to the largest value (B_4_). These parameters are all orthogonal to L and distributed in four perpendicular directions. The values of the steric parameters employed in this work were obtained from the literature (28,29,33).

Table 1 displays the physicochemical parameters used in the present work. These data are presented in the Material and Methods section, and not in the Results, because they were either calculated previously (30) or obtained in the literature (28,29,31-33).

**Table 1:** Physicochemical parameters of procainamide(PA) and N-acetylprocainamide (NAPA).

	Lipophilicity			Electronic		Steric				
	log P	log D	π	pKa	σ_p_	L	B_1_	B_2_	B_3_	B_4_
PA	1.10	-1.18	-1.23	9.4	-0.66	2.93	1.50	1.50	1.84	1.84
NAPA	1.29	-0.95	-0.97	8.3	0.00	5.15	1.50	1.90	1.94	3.61

log P: logarithm of the octanol/water partition coefficient; log D: logarithm of the lipid distribution coefficient: log P - (1+ 10^(pKa-pH)^); π: descriptor of the lipophilicity of the substituent in the benzene ring; σ_p_: Hammett's constant of aromatic p-substituents; L: length (Å) of the substituents; B_1_, B_2_, B_3_, and B_4_: widths (Å) of the substituents, from smallest (B_1_) to largest (B_4_)

### Statistics

In the analysis, different cells were pooled into one of the different experimental situations encompassed by the present work, i.e., control and 0.8, 3.5, and 7.0 mM of either PA or NAPA. This procedure assured independence among samples and resulted in a large number of samples, which were statistically managed using a multiple comparison statistical test (34). More specifically, statistical analyses were performed at the 5% level of significance using ANOVA followed by Tukey's test. This approach permitted the statistical comparison of pairs of sample averages (34) of interest within the aim of the present work. MINITAB^®^, provided by the Electronic Computing Center (CCE) of the University of São Paulo (Brazil), was used for statistical analysis. The data are reported as means±SE.

## Results

**Figure 2:**
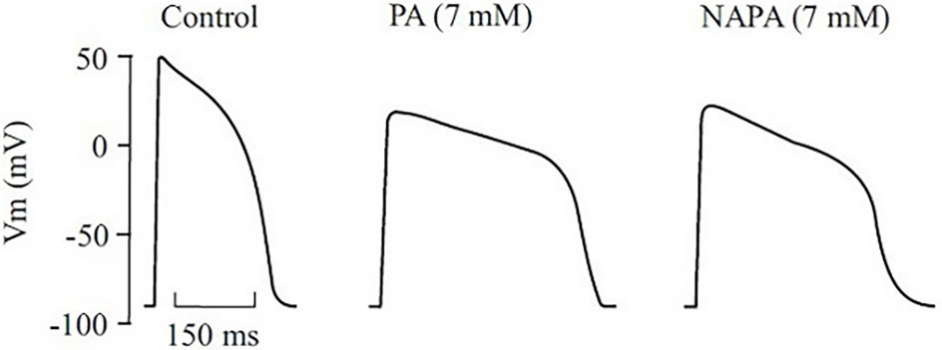
Digitized recordings of typical action potentials recorded intracellularly in the right papillary muscle of a guinea pig. In the control situation, the values of the corresponding electrophysiological variables were as follows: maximum upstroke velocity of the action potential (Vmax): 134 V/s; half-decay time of the action potential (HDT): 187 ms; and resting membrane potential (RMP): -83 mV. In the presence of procainamide (PA, 7 mM) the values were: Vmax: 42 V/s, HDT: 578 ms, and RMP: -86 mV. In the presence of N-acetylprocainamide (NAPA, 7 mM) the values were: Vmax: 48 V/s, HDT: 420 ms, and RMP: -82 mV. Vm: membrane potential.

Figure 2 exemplifies typical individual recordings of action potentials under our experimental conditions. Of note, both PA and NAPA affected Vmax and HDT without significantly affecting the resting membrane potentials.

**Figure 3:**
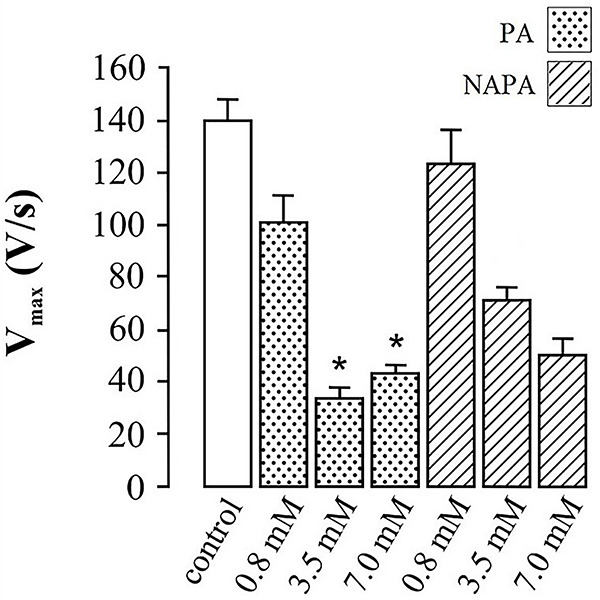
Effects of procainamide (PA) and N-acetylprocainamide (NAPA) on the maximum upstroke velocity (Vmax) of action potentials in guinea pig papillary muscle. Data are reported as means±SE of Vmax of action potentials recorded in 5-8 cells under each condition (*P<0.05, ANOVA followed by Tukey's test) for the difference in Vmax induced by PA and NAPA at similar concentrations of 3.5 or 7.0 mM.

**Figure 4:**
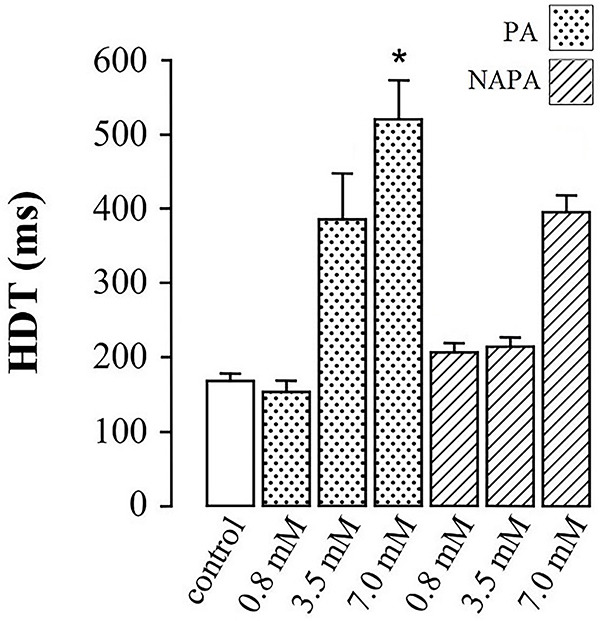
Effects of procainamide (PA) and N-acetylprocainamide (NAPA) on the half-decay time (HDT) of action potentials in guinea pig papillary muscle. Data are reported as means±SE of HDT of action potentials recorded in 5-8 cells for each condition (*P<0.05, ANOVA followed by Tukey's test) for difference in HDT induced by PA and NAPA at a similar concentration of 7.0 mM.

Figures 3 and 4 present the data concerning the effects of PA and NAPA, at several concentrations (0.8, 3.5, and 7.0 mM), on the electrophysiological variables studied, namely Vmax (Figure 3) and HDT (Figure 4).

Regarding the comparative effects of PA and NAPA on Vmax, Figure 3 illustrates that at the concentrations of 3.5 and 7.0 mM both PA and NAPA decreased Vmax significantly. It should be further noted that PA and NAPA differed significantly (P<0.05, ANOVA followed by Tukey's test), at both concentrations, in their ability to decrease Vmax (Figure 3), PA having shown a higher potency over NAPA on Vmax blockade.

Concerning the comparative effects of PA and NAPA on HDT, Figure 4 shows that both PA and NAPA increased HDT significantly at the concentration of 7.0 mM. Furthermore, PA and NAPA differed significantly (P<0.05, ANOVA followed by Tukey's test) in their ability to increase HDT, at a concentration of 7.0 mM, indicating a higher potency of PA over NAPA with regard to the prolongation of the action potential.

In summary, PA had stronger effects on HDT and Vmax than NAPA.

## Discussion

This study encompassed two steps. First, the effects of PA and NAPA on Vmax and HDT of the cardiac action potential were systematically and comprehensively compared using electrophysiological techniques. Second, these findings were comparatively scrutinized, in molecular terms, using lipophilicity and electronic and steric physicochemical parameters (28-33).

### Effects of PA and NAPA on Vmax and HDT of the cardiac action potential

The effects of PA and NAPA on Vmax and HDT of the cardiac action potential were interpreted assuming that modifications of Vmax reflect changes of cardiac Na^+^ channels whereas modifications of HDT reflect the drugs' blocking effects on cardiac K^+^ channels, especially cardiac hERG K^+^ channels, considering their physiological importance and that these channels are the most sensitive to blockade among the cardiac K^+^ channel subtypes (1,2).

While PA decreased Vmax at all three concentrations tested (Figure 3), it only increased HDT at the intermediate and highest concentrations (Figure 4). Thus, under the aforementioned assumptions PA blocked cardiac Na^+^ channels at a lower concentration and it also blocked cardiac hERG K^+^ channels at higher concentrations, as expected of a class IA antiarrhythmic drug (1,2).

In addition, NAPA had weaker effects on Vmax and HDT than PA (Figures 3 and 4). It should be emphasized that NAPA decreased Vmax at a concentration that had no effects on HDT (Figures 3 and 4, comparing intermediate concentrations). In other words, Vmax was more sensitive to NAPA than HDT, under the experimental conditions used. Thus, based on the aforementioned assumptions NAPA was demonstrated to interact with Na^+^ channels and affect these channels more potently than it affected hERG K^+^ channels. Because NAPA affected both Vmax and HDT, this compound cannot be considered a “pure” class III antiarrhythmic drug under the experimental conditions used in this study, because this drug class is expected to affect exclusively HDT of the action potential (1,2).

Therefore, it should be further stated that although we clearly detected the effects of NAPA on Vmax (Figure 3), this was not demonstrated in previous studies (4-7). The findings of these previous studies were, of course, absolutely correct. In our view, the discrepant results are attributable to species characteristics which can affect cardiac electrophysiology and, consequently, the cardiac pharmacology (8). Within this line of thought, the four previously mentioned studies (4-7) that found no effects of NAPA on Vmax were all performed in dogs. On the contrary, our study was performed in guinea pigs, supporting previous findings of different effects of NAPA on Vmax when species other than dogs and additional experimental approaches are considered.

Thus, we previously demonstrated that NAPA could block Na^+^ currents recorded by patch-clamp techniques in mouse cardiac myocytes (9). In addition, it has been reported that NAPA inhibits the binding of radioactive batrachotoxin to rat cardiac myocytes (35). The binding sites to batrachotoxin are believed to represent Na^+^ channels, considering that this toxin is a specific ligand for these channels (35). Further evidence of a possible action of NAPA on Na^+^ cardiac channels was provided by experiments using the electrically stimulated isolated atria to evaluate the refractory period of cardiac muscle. Thus, the available literature indicates that this refractory period, which is mainly modulated by the inactivation of cardiac Na^+^ channels, is increased by NAPA in the rat (36) and rabbit atria (37). The results of these studies are best understood by assuming that NAPA is active on cardiac Na^+^ channels.

Taken together, the aforementioned findings indicate that the effect of NAPA on Vmax, observed in this work (Figure 3), which reflects its effects on Na^+^ channels, is a general trend that is exceptionally not observed in dogs, the species previously used in studies in which no effects of this compound on Vmax were observed (4-7).

Thus, the results of this electrophysiological study do not support the previously reported concept that NAPA is a paradigmatic pure class III antiarrhythmic drug (1,2). In fact, under the experimental conditions used in this study, NAPA's pharmacological profile more strongly resembled that of a class IA antiarrhythmic drug, because NAPA affected Vmax at a lower concentration than it affected HDT (Figures 3 and 4). To the best of our knowledge, this feature of the pharmacology of NAPA has not been described previously and it constitutes a pharmacological feature that is worth studying from a molecular viewpoint, as approached in the next section.

### Molecular basis underlying the differential potency of PA and NAPA

In the previous section, an electrophysiological/pharmacological study suggested that NAPA was less potent than PA regarding their effects on cardiac Na^+^ and hERG K^+^ channels. Thus, the mechanism supporting these differential effects should be explored.

In pursuit of an answer, we considered the available data regarding the chemical structure of binding sites for blockers of either cardiac Na^+^ (14-19) or hERG K^+^ channels (20-24). Interestingly, both channels harbor binding sites featuring aromatic amino acids, tyrosine, and phenylalanine, which play key roles in the binding of blockers of those channels. The identity of these amino acids was determined by site-directed mutation studies. More specifically, in Na^+^ channels, the positions of the tyrosine and phenylalanine are, respectively, 1771 and 1764 (14-19), whereas in hERG K^+^ channels the amino acids are located at positions 652 and 656, respectively (20-24).

Considering the chemical structures of binding sites in cardiac Na^+^ and hERG K^+^ channels, as previously indicated, the possible mechanisms for the interaction of PA and NAPA with these binding sites were evaluated, comparatively, in light of the physicochemical parameters of these drugs. To ensure a comprehensive approach, the employed physicochemical parameters covered a large spectrum of properties, i.e., lipophilicity and electronic and steric parameters (28-33). Their numerical values are reported in Table 1, in the Material and Methods section. It is expected that this approach will reveal the molecular basis of the reason behind PA being more potent than NAPA.

Considering the lipophilicity parameters (log P, log D, and π), Table 1 shows that the values corresponding to NAPA were all greater than those corresponding to PA. This indicated that NAPA was more lipophilic than PA, as previously reported (4). Thus, the higher potency of PA over NAPA, found in this work, was theoretically unexpected, given the known positive correlation between potency and lipophilicity, regarding Na^+^ (14-19) and hERG K^+^ channel blockers (20-24). Therefore, the comparative pharmacology of PA and NAPA described in this work appears to violate this correlation. Clarification of this phenomenon, at the molecular level, is therefore required.

It should be noted that lipophilicity parameters modulate hydrophobic interactions that appear to explain the increase in potency with increasing lipophilicity in most circumstances, involving blockers of Na^+^ (10-12,14-19) and hERG K^+^ channels (20-24). However, hydrophobic interactions can be hindered by the size of the acting molecule, because the target sites for hydrophobic interactions are sometimes structured, for example, in the form of hydrophobic pockets, which set limits on the size of an interacting molecule. To delve deeper into this issue, the steric parameters of PA and NAPA were examined.

As presented in Table 1 (right columns), four of the five STERIMOL steric parameters were larger for NAPA. The data indicated that the larger size of NAPA was concomitantly linked to increased lipophilicity (left columns). Therefore, it is fair to suppose that, in the case of NAPA, competition between the increase in size and the increase in lipophilicity existed, in which steric hindrance offset the increase in lipophilicity. This is one possible reason why NAPA was less potent than PA despite being more lipophilic.

After examining lipophilicity and steric physicochemical parameters, electronic parameters were examined next to determine whether additional binding mechanisms of PA and NAPA to cardiac Na^+^ or hERG K^+^ channels contributed to the higher potency of PA over NAPA. Within mechanisms that are modulated by electronic parameters, the possible participation of aromatic-aromatic interactions was examined because such interactions have already been considered to participate in the binding of other blockers to Na^+^ (14-19) and hERG K^+^ channels (20-24).

Aromatic-aromatic interactions (38) (also denoted π-π or π-stacking interactions) could indeed explain the present findings, in view of the possible interactions between the aromatic ring of either PA or NAPA (Figure 1A and B) and the aromatic ring of tyrosine or phenylalanine residues found in the binding sites of either Na^+^ (14-19) or hERG K^+^ channels (20-24). Within this context, it should be noted that aromatic-aromatic interactions are influenced by the reactivity of the involved aromatic rings (39). For PA and NAPA, this reactivity can be described by examining Hammett's σ_p_. This value was negative for PA and null for NAPA (Table 1, middle columns). The negative value of Hammett's σ_p_ of the amino group in PA indicates that this is an electron-donating group (39), and it is therefore able to induce a redistribution of the charge in the substituted molecule, increasing its electron transferring capacity (39). This does not apply to NAPA, as Hammett's σ_p_ of its acetoamide group was null (Table 1, middle columns), which implies lower reactivity of the aromatic ring in NAPA, rendering the compound less capable than PA of undergoing aromatic-aromatic interactions with binding sites in cardiac Na^+^ and hERG K^+^ channels. Therefore, the weaker aromatic-aromatic interactions, in the case of NAPA, represent another possible explanation for its lower pharmacological potency than PA.

Another binding mechanism that must be considered in this work is the cation π-interaction, because this mechanism is apparently effective for a number of blockers of Na^+^ (14-19) and hERG K^+^ (20-24) channels. The mechanism should be considered in this work because of the amino-termini of PA and NAPA (Figure 1A and B). More specifically, because the amino-terminus is ionizable, the cationic species of PA and NAPA can undergo cation-π interactions (40) with the previously mentioned aromatic amino acids present in the binding sites of both cardiac Na^+^ (14-19) and hERG K^+^ channels (20-24). Regarding cation-π interactions, a physicochemical parameter, one molecular electronic property, that must be considered is pKa, as it can influence cation-π interactions. The pKa was larger for PA than for NAPA (Table 1, middle columns). Thus, under similar experimental conditions, a larger number of protonated species were present for PA than for NAPA, allowing PA to have more cation-π interactions between the protonated tertiary amine (Figure 1A and B) and the aromatic tyrosine and phenylalanine residues residing in the binding sites of cardiac Na^+^ (14-19) and hERG K^+^ channels (20-24).

In summary, the electrophysiological analysis revealed that NAPA behaved as a class IA antiarrhythmic drug, at a qualitative level, as opposed to a pure class III antiarrhythmic drug as shown in other studies, in dogs (4-7). This discrepancy is probably the result of species-dependence, a feature known to markedly affect the physiology/pharmacology of cardiac muscle (8). At a quantitative level, NAPA was less potent than PA. The analysis employed to disclose the molecular features responsible for the lower potency of NAPA compared to PA, based on the physicochemical parameters of these drugs, indicated that steric hindrance of NAPA effects was a possible, but not the only explanation for the lower potency of NAPA. In fact, aromatic-aromatic and cation-π interactions, which were more common for PA than for NAPA, also contributed to this finding and, together with steric hindrance, counteracted the higher lipophilicity of NAPA, which was expected to make it more potent than PA.

## References

[1] Armstrong EJ, Clapham DE, Golan DE, Tashjian AH, Armstrong EJ, Armstrong AW (2017). Pharmacology of cardiac rhythm.

[2] Knollmann BC, Roden DM, Brunton LL, Hilal-Dandan R, Knollman B (2018). Goodman & Gilman’s The Pharmacological Basis of Therapeutics, 13th ed.

[3] Obach S (2013). Pharmacologically active drug metabolites: impact on drug discovery and pharmacotherapy. Pharmacol Rev.

[4] Bagwell EE, Walle T, Drayer DE, Reidensberg MM, Pruett JK (1976). Correlation of the electrophysiological and antiarrhythmic properties of the N-acetyl metabolite of procainamide with plasma and tissue drug concentrations in the dog. J Pharmacol Exp Ther.

[B05] Dangman KH, Hoffman BF (1981). *In vivo* and *in vitro* antiarrhythmic and arrhythmogenic effects of N-acetylprocainamide. J Pharmacol Exp Ther.

[B06] Wu KM, Hoffman BF (1987). Effect of procainamide and N-acetylprocainamide on atrial flutter: studies *in vivo* and *in vitro*. Circulation.

[7] Coyle JD, Carnes CA, Schaal SF, Muir WW (1992). Electrophysiologic interactions of procainamide and N-acetylprocainamide in isolated canine cardiac Purkinge fibers. J Cardiovasc Pharmacol.

[8] Edwards AG, Louch WE (2017). Species-dependent mechanisms of cardiac arrhythmia: a cellular focus. Clin Med Insights Cardiol.

[9] Sigler W, Oliveira AC, Benndorf K (2002). Differential apparent affinity of procainamide and N-acetylprocainamide for cardiac Na^+^ channels. Plenary Lectures, Oral Sessions, Poster Sessions and Symposia. Pflug Arch Eur J Phy.

[10] Courtney KR, Strichartz GR, Strichartz GR (1987). Local anesthetics.

[B11] Baca QJ, Schulman JM, Strichartz GR, Golan DE, Tashjian AH, Armstrong EJ, Armstrong AW (2017). Local anesthetic pharmacology.

[12] Catterall WA, Mackie K, Brunton LL, Hilal-Dandan R, Knollman B (2018). Goodman & Gilman's The Pharmacological Basis of Therapeutics.

[13] Juhász V, Hornyk T, Benák A, Nagy N, Husti Z, Pap R (2018). Comparison of the effects of I_K,Ach_, I_Kr_ and I_Na_ block in conscious dogs with atrial fibrillation and on action potentials in remodeled atrial trabeculae. Can J Physiol Pharmacol.

[14] Ragsdale DS, McPhee JC, Scheuer T, Catterall WA (1994). Molecular determinants of state-dependent block of Na^+^ channels by local anesthetics. Science.

[B15] Qu Y, Rogers J, Tanada T, Scheuer T, Catterall WA (1995). Molecular determinants of drug access to the receptor site for antiarrhythmic drugs in the cardiac Na^+^ channel. Proc Natl Acad Sci USA.

[B16] Ragsdale DS, McPhee JC, Scheuer T, Catterall WA (1996). Common molecular determinants of local anesthetic, antiarrhythmic, and anticonvulsant block of voltage-gated Na+ channels. Proc Natl Acad Sci USA.

[B17] Yarov-Yarovoy V, Brown J, Sharp EM, Clare JJ, Scheuer T, Catterall WA (2001). Molecular determinants of voltage-dependent gating and binding of pore-blocking drugs in transmembrane segment IIIS6 of the Na^+^ channel α subunit. J Biol Chem.

[B18] Yarov-Yarovoy V, McPhee JC, Idsvoog D, Pate C, Scheuer T, Catterall WA (2002). Role of amino acid residues in transmembrane segments IS6 and IIS6 of the Na^+^ channel α subunit in voltage-dependent gating and drug block. J Biol Chem.

[19] Tikhonov DB, Zhorov BS (2017). Mechanism of sodium channel block by local anesthetics, antiarrhythmics and anticonvulsants. J Gen Physiol.

[20] Mitcheson JS, Chen J, Lin M, Culberson C, Sanguinetti MC (2000). A structural basis for drug-induced long QT syndrome. Proc Natl Acad Sci USA.

[B21] Fernandez D, Ghantal A, Kauffman GW, Sanguinetti MC (2004). Physicochemical features of the hERG channel drug binding site. J Biol Chem.

[B22] Sanguinetti MC, Mitcheson JS (2005). Predicting drug-hERG channel interactions that cause acquired long QT syndrome. Trends Pharmacol Sci.

[B23] Farid R, Day T, Friesner RA, Pearlstein RA (2006). New insights about HERG blockade obtained from protein modeling, potential energy mapping, and docking studies. Bioorgan Med Chem.

[24] Wacker S, Noskov SY, Perissinotti LL (2017). Computational models for understanding of structure, function and pharmacology of the cardiac potassium channel. Kv11.1 (hERG). Curr Top Med Chem.

[25] Sloan RC, Rosenbaun M, O'Rourke D, Oppel K, Frasier CR, Waston CA (2011). High doses of ketamine-xylazine anesthesia reduce cardiac ischemia-reperfusion injury in guinea pigs. J Am Assoc Lab Anim Sci.

[26] AVMA Guidelines for the euthanasia of animals (2020). AVMA.

[27] Baso ACZ, Serra CSM, Oliveira AC (2011). Relative contribution of pre- and post-synaptic effects to the neostigmine-induced recovery of neuromuscular transmission blocked by vecuronium. Fundam Clin Pharmacol.

[28] Kubinyi H (1993). QSAR: Hansch analysis and related approaches.

[B29] Patrick GL (2013). An introduction to medicinal chemistry.

[B30] (2005). ACD/Labs Software Version 9.0. [Computer program].

[B31] Weily HS, Genton E (1972). Pharmacokinetics of procainamide. Arch Intern Med.

[B32] Matusik E, Gibson TP (1975). Fluorometric assay for N-Acetylprocainamide. Clin Chem.

[33] Verloop A, Hoogenstraaten W, Tipker J, Ariëns EJ (1976). Drug design.

[34] Fisher LD, Van Belle G (1993). Biostatistics - A methodology for the health sciences.

[35] Sheldon R, Thakore E, Wilson L, Duff H (1994). Interaction of drug metabolites with the class I antiarrhythmic drug receptor on rat cardiac myocytes. J Pharmacol Exp Ther.

[36] Refsun H, Frislid K, Lunde PKM (1975). Effects of N-acetylprocainamide as compared with procainamide in isolated rat atria. Eur J Pharmacol.

[37] Minchin RF, Ilett KF, Paterson JW (1978). Antiarrhythmic potency of procainamide and N-acetylprocainamide in rabbits. Eur J Pharmacol.

[38] Burley SK, Petsko GA (1985). Aromatic-aromatic interaction: a mechanism of protein structure stabilization. Science.

[39] Durand-Niconoff JS, Cruz-Kuri L, Cruz-Sánches JS, Matus MH, Ramos-Morales FR (2012). Relationship between local reactivity indices and the Hammett constant for isatoic anhydride and its derivatives. Int J Quantum Chem.

[40] Dougherty DA (1996). Cation-π interactions in chemistry and biology: a new view of benzene, Phe, Tyr and Trp. Science.

